# COVID-19 and Superimposed Aspergillosis in a Dual Organ Recipient with Diagnosed B-Cell Lymphoproliferative Disorder: A Rare Case Report and Literature Review

**DOI:** 10.3390/diseases13100339

**Published:** 2025-10-14

**Authors:** Vidna Karadžić-Ristanović, Jelena Pavlović, Voin Brković, Ana Bontić, Marko Baralić, Dragan Vasin, Maja Životić, Novica Boričić, Darko Antić, Vojin Vuković, Milan Radović

**Affiliations:** 1 Clinic for Nephrology, University Clinical Center of Serbia, Pasterova 2, 11000 Belgrade, Serbia; jelena.pavlovic@med.bg.ac.rs (J.P.); voin.brkovic@med.bg.ac.rs (V.B.); ana.bontic@med.bg.ac.rs (A.B.); milan.radovic@med.bg.ac.rs (M.R.); 2 Faculty of Medicine, University of Belgrade, Dr Subotića Starijeg 8, 11000 Belgrade, Serbia; dragan.vasin@med.bg.ac.rs (D.V.); maja.zivotic@med.bg.ac.rs (M.Ž.); novica.boricic@med.bg.ac.rs (N.B.); darko.antic@med.bg.ac.rs (D.A.); vojin.vukovic@med.bg.ac.rs (V.V.); 3 Emergency Radiology Department, Center for Radiology, University Clinical Center of Serbia, Pasterova 2, 11000 Belgrade, Serbia; 4 Institute of Pathology, Faculty of Medicine, University of Belgrade, Dr Subotića Starijeg 1, 11000 Belgrade, Serbia; 5 Clinic for Hematology, University Clinical Center of Serbia, Dr Koste Todorovića 2, 11000 Belgrade, Serbia

**Keywords:** COVID-19, aspergillosis, kidney transplantation, B-cell lymphoma

## Abstract

Post-transplant lymphoproliferative disorder (PTLD) poses significant risks following organ transplantation, characterized by potential aggressiveness. This report aims to discuss a case of PTLD presenting as B-cell large-cell lymphoma (DLBCL) post kidney and pancreas transplantation. A 44-year-old female with type 1 diabetes underwent simultaneous cadaver kidney and pancreas transplantation. She presented with fever, night sweats, and weakness, revealing multiple lesions on CT, including in transplanted and native kidneys and pancreas. A biopsy of the transplant kidney confirmed PTLD, DLBCL subtype, with complex immunohistochemical findings. Chemotherapy (R-CHOP) was initiated but complicated by bowel perforation necessitating surgery and antibiotics, transplant renal vein thrombosis, pyelonephritis, and neutropenia. Despite the complications, the normal function of the transplanted kidney was maintained, which made it possible to implement the standard chemotherapy protocol. This case underscores the diagnostic challenges and therapeutic complexities of PTLD, specifically DLBCL, in transplant recipients. The co-infection of COVID-19 and aspergillosis in a multiple immunocompromised patient indicated a possible rapid course of the disease with global respiratory insufficiency and a fatal outcome despite all applied therapeutic modalities.

## 1. Introduction

Post-transplant lymphoproliferative disorder (PTLD) represents a severe and often insidious complication that can occur after kidney transplantation, characterized by its potential for aggressive dissemination [1]. PTLD predominantly arises in the context of Epstein–Barr virus (EBV) infection and is most frequently observed within the first year following transplantation. In planning for transplants and the treatment of recipients, it is essential to strike a balance between preventing certain complications such as transplant rejection via the utilization of immunosuppressive agents, and the potential risk of infectious complications and PTLD [2,3]. High-dose immunosuppression has been linked to an increased risk of PTLD; the clinical manifestations of this condition can vary significantly, ranging from localized lesions to widespread disease, and may mimic benign disorders [4].

The incidence of PTLD varies depending on factors such as the type of organ transplanted and the immunosuppressive regimen employed. A study utilizing data from the French kidney transplant registry, which followed adult patients prospectively from 1998 to 2007, reported a 1% incidence of PTLD at 5 years, increasing to 2.1% at 10 years post-transplant [5]. The likelihood of PTLD decreased with more recent transplants. Similarly, a Canadian single-center cohort study involving 1642 kidney transplant recipients from 2000 to 2012 documented a PTLD incidence rate of 0.18 cases per 100 person-years, with EBV mismatch between donor and recipient identified as a significant risk factor [6]. Key risk factors for PTLD include the EBV serostatus of the donor and recipient (with PTLD more common in EBV-seropositive donors and EBV-seronegative recipients), the intensity and duration of immunosuppressive therapy, and the status of EBV infection.

Although PTLD is relatively uncommon, there remains a significant gap in the literature regarding optimal management strategies. Accurate diagnosis relies on a thorough histopathological assessment, complemented by molecular analyses of tissue samples. Monomorphic PTLD represents the predominant subtype, accounting for approximately 76–83% of cases, with diffuse large B-cell lymphoma (DLBCL) being the most frequent histological variant, comprising nearly 79% of monomorphic PTLD. Renal allograft involvement is observed in about 17% of cases, emphasizing the importance of including PTLD in the differential diagnosis of graft dysfunction [7].

Data from Florida indicated that among transplant patients with COVID-19, 3.8% developed fungal co-infections, with the majority related to aspergillosis. These infections were typically diagnosed around 21 days after the initial COVID-19 diagnosis, and the associated mortality was alarmingly high at 73.9%. Key risk factors for aspergillosis in transplant patients with COVID-19 include prolonged viral shedding, lymphocytopenia, lack of vaccination, and the use of immunosuppressive agents (especially corticosteroids) [8].

Against this background, we report a rare and clinically significant case of PTLD manifesting as DLBCL in a patient following combined kidney and pancreas transplantation, further complicated by COVID-19 and invasive aspergillosis. The presentation of this case aims to highlight the diagnostic and therapeutic challenges arising from the convergence of post-transplant lymphoproliferative disease and severe infections in the setting of multiple layers of immunocompromise, emphasizing the need for heightened clinical awareness and multidisciplinary management. However, despite all applied therapeutic modalities, the outcome could be fatal, as illustrated by the current case.

## 2. Case Presentation

A 44-year-old female patient was admitted to the Clinic of Nephrology, University Clinical Center of Serbia, with elevated body temperature (40.2 °C) accompanied by night sweats and general weakness. In the laboratory, analyses found worsening transplant kidney function with an increase in serum creatinine (sCr) values (180 μmol/L, 59–104 μmol/L) and inflammation parameters (C-reactive protein (CRP) 275 mg/L, 0–8 mg/L). The patient had had a history of type 1 diabetes mellitus since the age of six, which led to diabetic kidney disease and end-stage kidney disease (ESKD). Consequently, she underwent simultaneous cadaver kidney and pancreas transplantation in January 2014 in NewYork-Presbyterian Hospital in the USA (https://www.nyp.org). Following this, she was treated with triple immunosuppressive therapy (Tacrolimus (TAC) 2 + 2 mg, Azathioprine 100 mg, Prednisone 5 mg). She was also treated with double antibiotic therapy (cefixime, ciprofloxacin), after which the symptoms subsided and normalization of inflammation parameters and kidney allograft function was achieved (sCr 99 μmol/L). Blood and urine cultures were negative, as well as PCR for EBV and CMV. The patient had had no previous COVID-19 infection and was not vaccinated against it.

**Figure 1:**
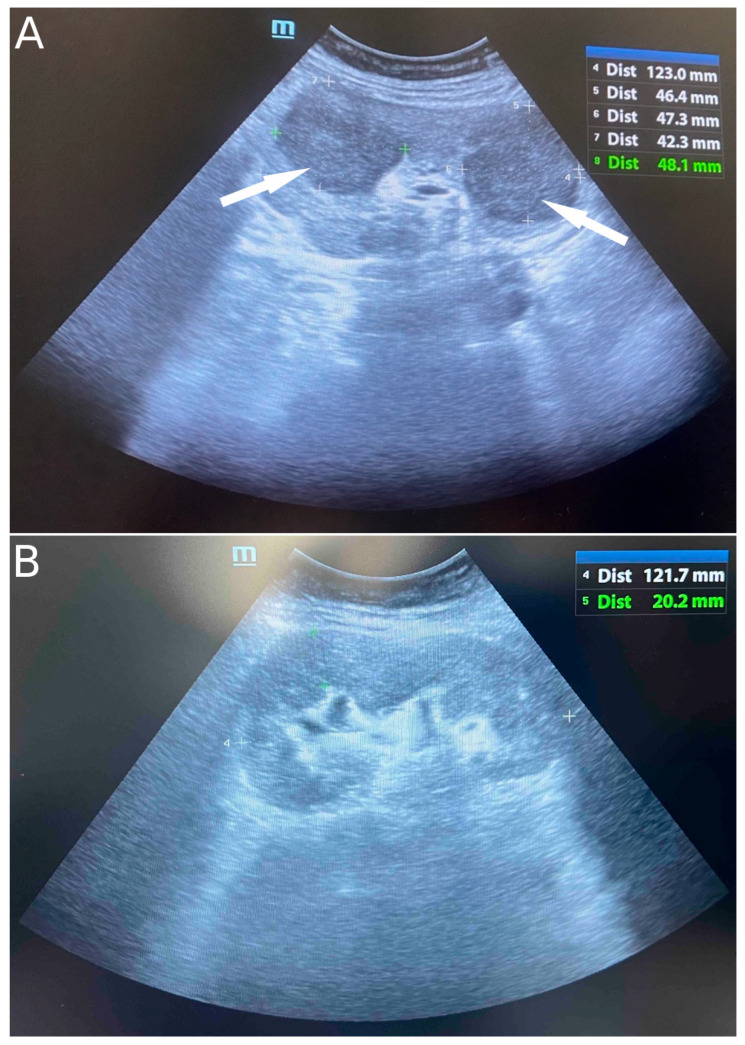
Ultrasound image of infiltrative circular lesions of transplant kidney lymphoma (indicated by arrows) (**A**) and normal ultrasound image of transplant kidney after six cycles of chemoimmunotherapy (**B**).

**Figure 2:**
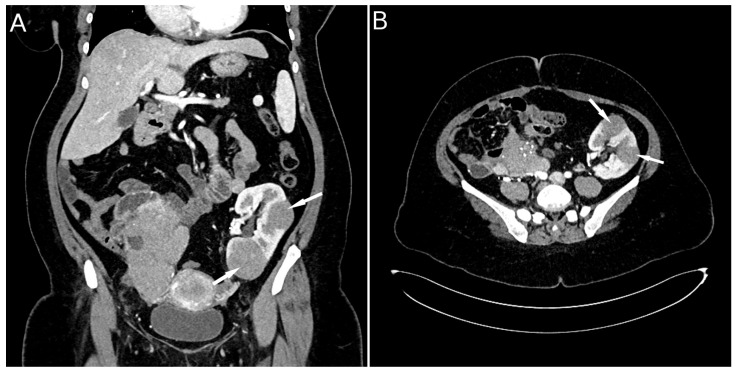
CT scan image: lesions in the transplanted kidney (**A**) and post-transplant lymphoma in a kidney allograft (**B**) (indicated by arrows).

On ultrasound of the transplant kidney, which was performed upon admission, three infiltrative circular lesions isoechoic with parenchyma were registered and raised the suspicion of the existence of an immunoproliferative disease. A CT scan was also performed using CT-GE Medical Systems, LCC, Revolution HD, Belgrade, Serbia, revealing three lesions in the transplanted kidney, a larger lesion in the body of the pancreas, and additional lesions in the native kidneys (Figure 1A and Figure 2). Additionally, a lesion was identified in the medial lobe of the right lung adjacent to the sixth rib cartilage.

**Figure 3:**
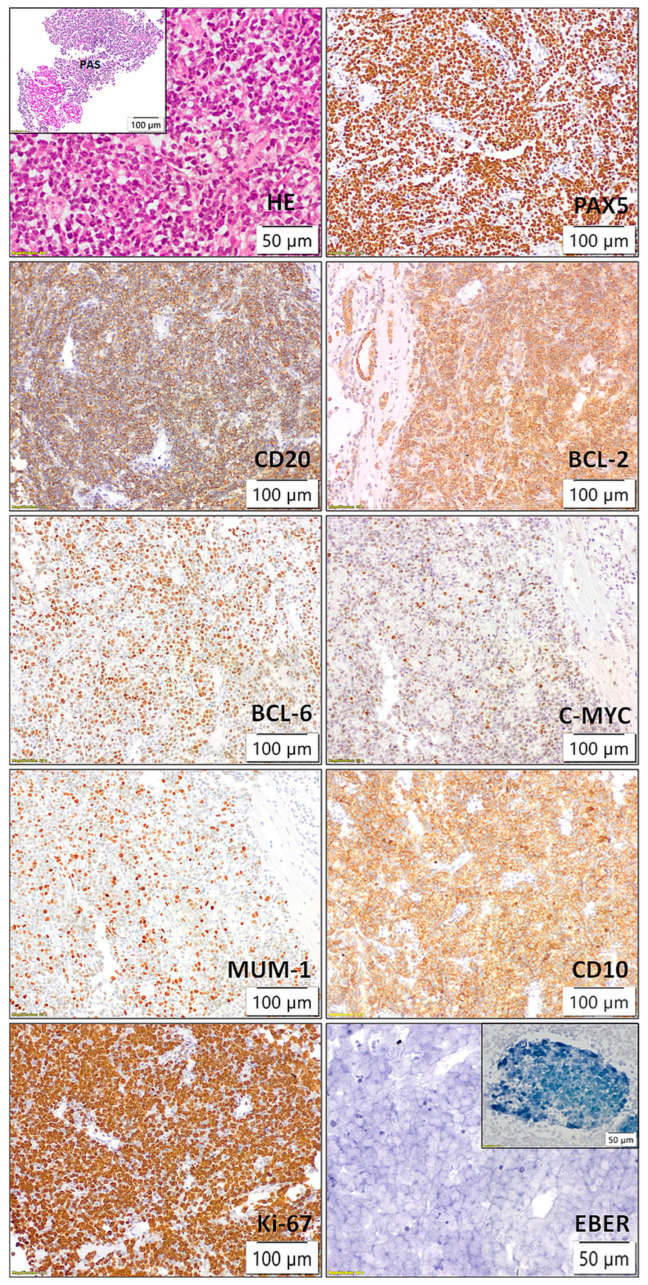
Histopathological and immunohistochemical findings of kidney transplant showing posttransplant lymphoproliferative disorder (PTLD), diffuse large B-cell lymphoma (DLBCL), germinal center B-cell (GCB) subtype, with a “double expressor” phenotype (BCL2 ≥ 50%, MYC ≥ 40%).

Following the identification of lesions, the patient underwent a biopsy of the lower pole of the transplanted kidney as the most accessible organ. Pathological and immunohistochemical analyses were conducted (Figure 3), which showed positive staining for CD20, Pax5, CD10, BCL6, BCL2, MUM1, c-myc, and lambda, while kappa, CD138, CD3, CD5, CD23, CyclinD1, Sox11, CD30, CD15, CD35, TdT, CKAE1/AE3, INSM1, and CD99 were negative. The Ki67 proliferation index was >90%. Two independent pathologists confirmed the diagnosis of monomorhic PTLD, specifically diffuse large B-cell lymphoma (DLBCL), GCB subtype, “double expressor” phenotype, given the positive staining for BCL 2 positivity in ≥50% of tumor cells and MYC positivity in ≥40% of tumor cells (Figure 3). In situ hybridization for EBER was negative (external EBER positive control is illustrated in upper right corner of the image) (Figure 3). FISH analysis using locus-specific split-signal probes for BCL2 (18q21.33, 3’ and 5’), BCL6 (3q27.3 3’ and 3q28 5’), and break-apart probe for C-MYC (8q24) was technically unsuccessful due to the limited amount of tumor tissue in the submitted material.

Taking into account initial CT findings and appropriate clinical and laboratory parameters at diagnosis, the disease was classified as Ann Arbor IV B E stage and high-intermediate risk group (3) according to the International Prognostic Index.

After receiving the HP and IHC findings of PTLD, a conversion was made: Tacrolimus (TAC) was changed with everolimus (Certican-mTOR inhibitor) 0.75 + 0.5 mg with dose correction according to target values (5–6 ng/mL). The antimetabolic agent was excluded, and the corticosteroid was continued in a low dose (Prednisone 5 mg).

A multidisciplinary team recommended chemoimmunotherapy according to the R-CHOP protocol (Rituximab, Cyclophosphamide, Doxorubicin, Vincristine, Prednisone) up to six cycles. During the chemoimmunotherapy treatment, the function of the allograft was normal but the patient developed a series of complications. After the third cycle of RCHOP, the patient developed a complication of small bowel perforation, which was surgically addressed with the creation of a terminal stoma on the anterior abdominal wall. After a recovery period of forty days, the patient resumed the fourth cycle of chemoimmunotherapy when non-occlusive transplant renal vein thrombosis was registered by a control CT, which was cured by the long-term use of anticoagulant therapy. After the fifth cycle of RCHOP, the clinical course was complicated by the appearance of pyelonephritis caused by Proteus mirabilis, leading to hospitalization at the Kidney Transplantation Center for treatment. After the sixth cycle of chemotherapy, the patient developed grade IV neutropenia with a neutrophil count of 0.5 × 10^9^/L, WBC 1.2 × 10^9^/L, which was treated with the application of Granulocyte colony stimulating factor (G-CSF).

After the completion of six cycles of RCHOP, the patient was in good general condition and afebrile, with maintenance of the normal function of the allograft. However, radiographic findings revealed active disease with infiltrates described in both the kidney and pancreas grafts, as well as in the lumbar and sacral vertebrae. An ultrasound image of the kidney graft is shown in Figure 1B. Salvage chemotherapy was indicated but never conducted due to post-treatment complications with an eventually fatal outcome.

**Figure 4:**
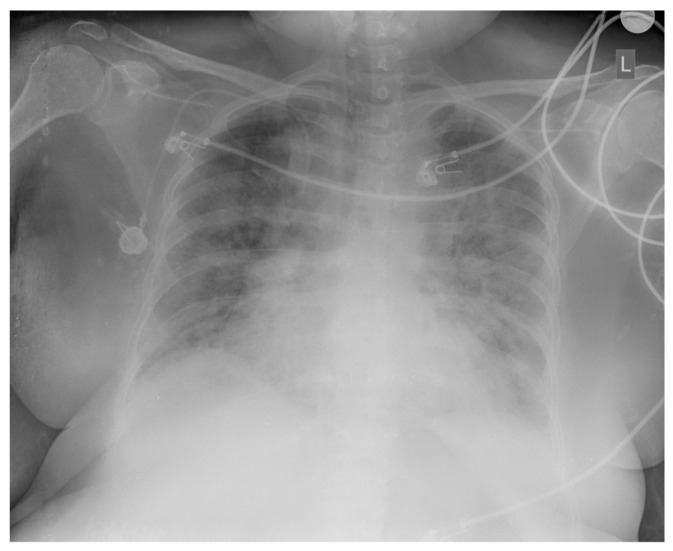
Chest X-ray: bilateral, peripheral, and lower-zone ground-glass opacities and consolidations consistent with COVID-19 pneumonia.

In October 2024, five months after the completed chemotherapy treatment, the patient was hospitalized at the Kidney Transplantation Center due to fever (38.5 °C), cough, and a high inflammation parameter value (CRP 123 mg/L). She was treated with double antibiotic therapy (meropenem, vancomycin). During hospitalization, a COVID-19 infection was diagnosed with a positive result from a PCR test for SARS-CoV-2 and consequent massive bilateral pneumonia (Figure 4), and the patient was treated with corticosteroid and oxygen therapy. All microbiological cultures were taken from the patient due to fever and known lymphoproliferative disease, and a positive PCR result for Aspergillus was obtained. Combined with the clinical picture and lung radiological findings, an infectious disease specialist diagnosed aspergillosis and caspofungin therapy was administered.

In a few hours, the patient developed high fever and severe global respiratory insufficiency as part of the co-infection (SARS-CoV-2 and apsergillosis), for which she was initially treated non-invasively (NIV), and then she was intubated and put on mechanical ventilation support using the CPAP method. The respiratory support parameters were PEEP 5, PS 10, FiO_2_ 70%. Despite all measures of intensive treatment, the patient’s fatal outcome was soon recorded.

The approval for publishing this case was granted by the Ethics Committee of the University Clinical Center of Serbia (No. 1264/20, from 4 July 2024).

## 3. Discussion

A rare case of PTLD, specifically DLBCL, presented in a patient who underwent simultaneous pancreas and kidney transplantation due to longstanding type 1 diabetes mellitus and diabetic kidney disease. The patient presented with elevated body temperature, a recognized sign of PTLD. According to Samant et al. [9], PTLD manifestations are heterogeneous, non-specific, and highly variable, presenting either as localized or disseminated disease. Common symptoms include malaise, fatigue, fever, and a mononucleosis-like syndrome. B-symptoms such as night sweats, weight loss, and lymphadenopathy are also prevalent [9]. PTLD can progress rapidly and may lead to compressive symptoms at the tumor site. High-risk patients, such as those with EBV IgG-positive donors and EBV-negative recipients, are particularly susceptible to PTLD, which can impair graft function [10]. Due to the diverse clinical presentation, a high index of suspicion for PTLD is essential, especially with increasing EBV PCR levels in post-transplant recipients, which heightens the likelihood of PTLD [11]. In EBV recipients, immunosuppression with mTORi+TAC has been associated with an increased risk of PTLD, death, and graft failure, while mycophenolate sodium (MMF)+cyclosporine (CsA) use is associated with a trend toward increased risk of rejection, lower PTLD risk, and similar risk for graft failure when compared with MMF+TAC [12]. CsA use or TAC avoidance in female patients younger than 45 years at transplantation postpones PTLD development [13].

### 3.1. PTLD Classification

Pathological analysis is essential for a definitive diagnosis of PTLDs. According to the International Consensus Classification (ICC) of Mature Lymphoid Neoplasms, published in 2022, as well as the fourth revised edition of the World Health Organization’s (WHO) classification of lymphoid malignancies from 2016, PTLDs are classified into four distinct categories based on their morphological, immunophenotypic, genetic, and clinical characteristics [14,15]. Accurate classification is crucial for directing appropriate management strategies [16]. The first category includes non-destructive PTLD (in previous classifications denominated as early lesions), such as plasmacytic hyperplasia, infectious mononucleosis-like reactions, and florid follicular hyperplasia, which are considered early and benign proliferations characterized by the preservation of normal lymph node architecture. The remaining categories are neoplastic and are defined by the presence of a lymphoid tumor combined with at least two of the following features: the disruption of tissue architecture by lymphoid proliferation, the presence of monoclonal or oligoclonal lymphoid cell populations, and widespread EBV infection [14]. Monomorphic PTLD is characterized by lymphomas of B- or T/NK cell origin, with diffuse large B-cell lymphoma being the most prevalent, though Burkitt lymphoma, plasma cell neoplasms, and other T/NK cell lymphomas may also occur. Polymorphic PTLD involves a heterogeneous lymphoid infiltrate that does not meet the criteria for specific B- or T/NK cell lymphomas. Lastly, Hodgkin lymphoma-like PTLD matches the diagnostic criteria for classical Hodgkin lymphoma and is the rarest form of PTLD [17]. In the most recent fifth edition of the WHO classification of lymphoid malignancies, PTLDs were designated within the group of entities called “lymphoid proliferations and lymphomas arising in the setting of immune deficiency/dysregulation”. Various conditions and circumstances in addition to the post-transplant state may underlie this group of lymphoproliferations, such as inborn error of immunity, HIV infection, autoimmune disease, iatrogenic/therapy-related conditions, and immune senescence [18].

### 3.2. Treatment Strategies

The management of PTLD involves several strategies distinct from those used for lymphoproliferative disorders in immunocompetent patients such as the National Comprehensive Cancer Network (NCCN) [19] and American Society of Transplantation (AST) guidelines (https://www.myast.org) [20]. Initial treatment typically includes reducing immunosuppression by lowering calcineurin inhibitors and discontinuing antimetabolic agents [21]. Rituximab, an anti-CD20 monoclonal antibody, is used for patients who do not respond adequately to reduced immunosuppression, either alone or in combination with chemotherapy. For those unresponsive to these measures, chemo(immuno)therapy, particularly the R-CHOP regimen, is employed [21,22]. Radiation therapy is used for localized disease or central nervous system involvement [23]. Additionally, adoptive immunotherapy utilizing EBV-specific cytotoxic T lymphocytes or donor lymphocyte infusion may be considered, though it carries a risk of graft-versus-host disease [24]. Amengual and Pro, in the “How I Treat” series, recommend a sequential approach to the treatment of monomorphic CD20+ PTLD, with initial 4-weekly administrations of rituximab monotherapy, while the escalation to chemoimmunotherapy is reserved for non-responding patients or high-risk patients in partial remission [25]. Given the multiple high-risk features of our patient (IPI 3, three extranodal sites involved) and poor treatment response to RIS and/or rituximab monotherapy of high-risk patients according to the literature data, we decided to administer chemoimmunotherapy from the beginning of hematological treatment [26,27,28].

The differential diagnosis of PTLD should account for its variable presentation, considering allograft rejection, opportunistic infections, and common infectious causes depending on the clinical context. The prognosis for PTLD has notably improved with the advent of rituximab and lymphoma-specific treatment regimens. An international trial demonstrated that sequential treatment with rituximab followed by CHOP chemotherapy resulted in a complete or partial response in 53 of 59 patients, with 40 achieving a complete response [26]. Retransplantation is feasible following PTLD treatment, but is only recommended after a minimum of one year [29].

### 3.3. COVID-19 Associated Complications

Kaplka et al. reported that COVID-19-associated pulmonary aspergillosis most often occurs within days/weeks after COVID-19 infection. The infection develops, on average, 18 days after SARS-CoV-2 infection, which was not the case in our patient, but occurred within 2/3 days [30]. Bhopalwala reported late manifestations of COVID-19-associated pulmonary aspergillosis within months after infection [31,32]. Risk factors for COVID-19-associated pulmonary aspergillosis are most often associated with age, active smoking, chronic respiratory disease, chronic kidney disease, and chronic corticosteroid therapy (CCT). Our presented patient had only CCT, while global kidney function was preserved.

COVID-19 infection is most often reported to have a major impact on the immune system during and several months after infection. In patients with co-infection, the immune system is compromised by functional impairment of natural killer cells and T cells. This has been revealed in patients with COVID-19 through reduced levels of its markers, NK-G2A and PD-1 [33]. The reduced fungicidal activity of neutrophils in patients with COVID-19 suggests that immune imbalance is an important risk factor for invasive fungal diseases, especially considering that the patient was a double transplant recipient and had been on immunosuppressive therapy for many years.

## 4. Conclusions

This case report details a rare instance of DLBCL as a PTLD in a 44-year-old female with a history of type 1 diabetes and simultaneous kidney and pancreas transplantation. Considering the manifestation of lymphoproliferative disease in the form of infiltrative changes on the transplanted kidney, an earlier diagnosis of DLBCL was possible. Due to the normal function of the transplanted kidney, it was possible to carry out necessary chemotherapy in full doses of drugs. Despite complications such as transplant renal vein thrombosis, bowel perforation, and neutropenia, the patient’s health improved with ongoing follow-up and treatment. Due to all of the above, co-infection with SARS-CoV-2 and aspergillosis can lead to rapid respiratory failure within a few hours of a positive PCR test and result in a fatal outcome. It is possible that prior immunization with an mRNA COVID-19 vaccine could have mitigated the severity of the infection.

## Data Availability

The raw data supporting the conclusions of this article will be made available by the authors on request.
